# The Effects of Landscape Modifications on the Long-Term Persistence of Animal Populations

**DOI:** 10.1371/journal.pone.0008932

**Published:** 2010-01-28

**Authors:** Jacob Nabe-Nielsen, Richard M. Sibly, Mads C. Forchhammer, Valery E. Forbes, Christopher J. Topping

**Affiliations:** 1 Department of Arctic Environment, National Environmental Research Institute, University of Aarhus, Roskilde, Denmark; 2 Centre for Integrated Population Ecology (CIPE), Department of Environmental, Social and Spatial Change, Roskilde University, Roskilde, Denmark; 3 School of Animal and Microbial Sciences, University of Reading, Reading, United Kingdom; 4 Department of Environmental, Social and Spatial Change, Roskilde University, Roskilde, Denmark; 5 Department of Wildlife Ecology & Biodiversity, National Environmental Research Institute, University of Aarhus, Rønde, Denmark; University of Bristol, United Kingdom

## Abstract

**Background:**

The effects of landscape modifications on the long-term persistence of wild animal populations is of crucial importance to wildlife managers and conservation biologists, but obtaining experimental evidence using real landscapes is usually impossible. To circumvent this problem we used individual-based models (IBMs) of interacting animals in experimental modifications of a real Danish landscape. The models incorporate as much as possible of the behaviour and ecology of four species with contrasting life-history characteristics: skylark *(Alauda arvensis)*, vole *(Microtus agrestis)*, a ground beetle *(Bembidion lampros)* and a linyphiid spider *(Erigone atra)*. This allows us to quantify the population implications of experimental modifications of landscape configuration and composition.

**Methodology/Principal Findings:**

Starting with a real agricultural landscape, we progressively reduced landscape complexity by (i) homogenizing habitat patch shapes, (ii) randomizing the locations of the patches, and (iii) randomizing the size of the patches. The first two steps increased landscape fragmentation. We assessed the effects of these manipulations on the long-term persistence of animal populations by measuring equilibrium population sizes and time to recovery after disturbance. Patch rearrangement and the presence of corridors had a large effect on the population dynamics of species whose local success depends on the surrounding terrain. Landscape modifications that reduced population sizes increased recovery times in the short-dispersing species, making small populations vulnerable to increasing disturbance. The species that were most strongly affected by large disturbances fluctuated little in population sizes in years when no perturbations took place.

**Significance:**

Traditional approaches to the management and conservation of populations use either classical methods of population analysis, which fail to adequately account for the spatial configurations of landscapes, or landscape ecology, which accounts for landscape structure but has difficulty predicting the dynamics of populations living in them. Here we show how realistic and replicable individual-based models can bridge the gap between non-spatial population theory and non-dynamic landscape ecology. A major strength of the approach is its ability to identify population vulnerabilities not detected by standard population viability analyses.

## Introduction

The relationship between landscape complexity and population dynamics is poorly understood, even though the spatial structure of populations is recognized to play a major role in their persistence [1], [2]. Landscapes can be managed to improve living conditions for animals by creating dispersal corridors, by ensuring that similar habitat patches are located close together or by altering the size of the patches [3], [4], [5], [6], [7]. One obvious management goal is to make it easier for animals to move among patches with complementing resources or to unoccupied high-quality patches [8], [9], thereby increasing the functional connectivity of the landscape [10], [11], [12], [13]. Hence, a key issue in conservation and landscape ecology has been to understand how the arrangement and size of habitat patches affects the dynamics and long-term persistence of species with different life histories [5], [11], [14], [15], [16], [17], [18], [19]. Specifically we need to understand the relative importance of corridors (linear patches that facilitate movement between main habitat patches), landscape configuration (spatial arrangement of patches) and composition (relative cover of patch types).

The impact of changes in landscape structure on population dynamics can be characterized in terms of changes in the equilibrium population size, *K*, and population return time, *φ*, i.e. time to recovery after disturbance. *φ* is defined as the reciprocal of return rate [20], [21], also known as the strength of density dependence [22], [23] (see Appendix S1). In fragmented landscapes [5] with few corridors or with habitat patches located far apart, subpopulations become isolated and less likely to be maintained through continuous immigration (the ‘rescue effect’ [7], [24]), and *K* is consequently reduced. Recovery from catastrophes may also be slower in fragmented landscapes because the recolonization of empty habitat patches takes longer, particularly in species with limited dispersal ability [25], [26].

Classical theory of population dynamics assumes a spatially homogeneous environment where individuals have equal access to resources [20], [22], [23], [27], [28], but a spatially explicit approach is needed to study the effects of landscape complexity [1], [5], [15], [18], [29]. The effects of landscape alterations on local population densities have been studied using spatially explicit reaction-diffusion models [6] and individual-based models (IBMs) (e.g. [14], [15], [30], [31]) based on simplistic landscapes. To our knowledge no study has hitherto attempted to develop conceptual models of how landscape alterations affect population dynamics for different kinds of animals in landscapes using realistic environmental settings. It is therefore high priority for ecology to investigate how ecologically different species respond to changes in complexity in contemporary landscapes. Here we study population characteristics using spatially explicit IBMs where the overall population dynamics emerge solely as a result of individuals' independent and autonomous site-specific behaviors [32], [33]. The approach permits us to get a unique insight into the link between landscape complexity and population dynamics by separating the effects of corridor availability, landscape configuration and composition using landscapes with modified patch shapes. We provide the first analysis of how changes in landscape structure influence the long-term dynamics (i.e. *K* and *φ*) of entire populations in realistic landscapes. This we do for four species embracing a range of different life-history characteristics (long- and short dispersing, fast- and slow reproducing) to test the predictions that *K* decreases and *φ* increases with increasing landscape fragmentation, particularly in short-dispersing species.

## Results

**Figure 1:**
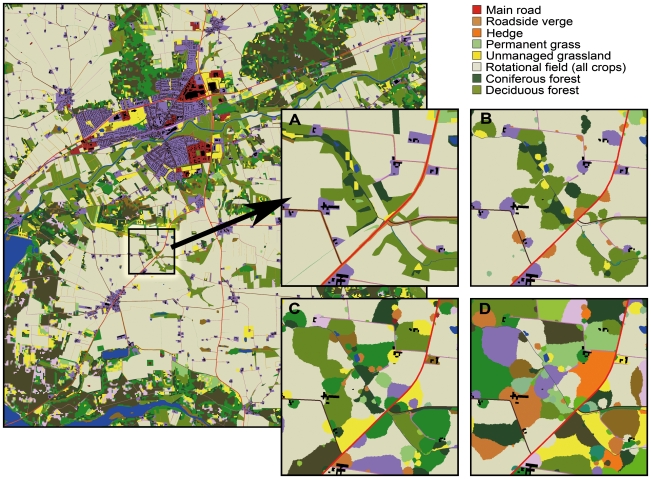
Landscape from Bjerringbro, Denmark (10×10-km). Insets show increasingly simplified landscapes used in simulations: (A) magnified portion of the original landscape; (B) landscape with homogenized patch shapes, but with unaltered patch sizes and locations. (C) randomly interchanged patch locations, patch shapes as in B. On the landscape scale (but not in the insets) the relative cover of each patch type is unaltered in A–C; (D) randomized patch locations and sizes. Potential dispersal barriers (roads, houses, lakes etc.) were maintained as in the original landscape throughout.

The population simulations were carried out in four different landscapes (a small part of each is shown in Fig. 1A–D). Reference simulations were obtained using a real agricultural landscape (main map in Fig. 1; 1A). Thereafter we progressively reduced landscape complexity by removing the constraints on patch arrangement and sizes imposed by human activities, soil types etc., thereby obtaining decreasingly structured landscapes. First, potential corridors were removed by homogenizing patch shapes (B). Next, patch arrangement was randomized by interchanging homogenized patches of similar sizes (C). Steps B–C resulted in alterations of landscape configuration without changing landscape composition. As similar patches at the same time became more separated, it therefore resulted in increasing landscape fragmentation *sensu* Fahrig [5]. In the final step (D) patch arrangement was randomized by interchanging homogenized patches irrespective of their sizes. This changed the relative cover of the different patch types (see Fig. S1) and the landscape composition was consequently altered.

**Figure 2:**
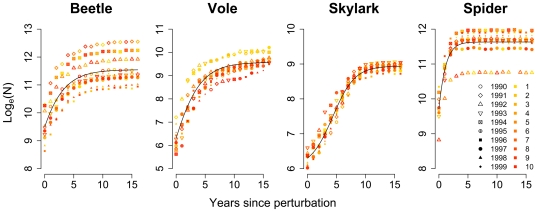
Population growth in the original 10×10-km landscape after 95% reductions in population size. Perturbations occurred every 17 years, and points have different colors depending on how many times perturbations had occurred. Weather years (i.e. the year that the weather data originated from) are indicated with different symbols. Curves were fitted using a three-parameter logistic model (four-parameter for skylark).

**Figure 3:**
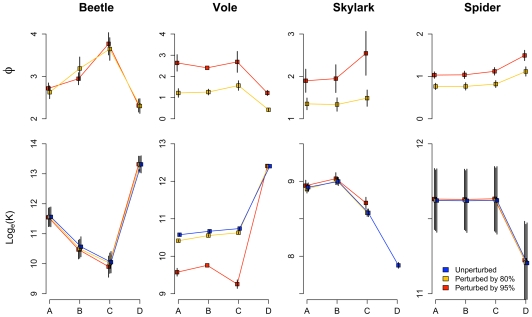
Return times (*φ*) and equilibrium population sizes (*K*) in four successively simplified landscapes, fitted as in Fig. 2. A–D are as in Fig. 1, with A being the original and most complex landscape. Colors indicate perturbation intensity. Error bars are 95% confidence intervals corresponding to variation among weather years. For vole perturbed by 95% no confidence intervals could be calculated in landscape B as the mixed model did not converge. No return times were calculated for unperturbed populations. For skylark the population did not increase logistically in landscape D so *K* and ***φ*** could not be calculated.

Yearly population number increased logistically with time following disturbances for beetle, vole, skylark and spider (Fig. 2). Except for the vole, the number (1–10) of times a population was consecutively perturbed by removing a large part of the population had no effect on asymptotic population sizes (*K*). The logistic growth fluctuated among years with different weather conditions so that *K* varied between years, especially in the beetle and spider populations. Mean values of *K* varied among landscapes of different complexity (Fig. 3). Across landscapes low *K* was associated with long return time *φ* for the short-dispersing species (*r* = −0.92 for beetle; *r* = −0.87 for vole; *P*<0.01 for both species) whereas the correlation was non-significant for skylark and spider. Increasing disturbance intensities (i.e. removing a larger part of the population) caused large increases in *φ*, especially for the relatively slow-reproducing vole and skylark.

Removal of potential dispersal corridors (transitions A to B in Figs. 1 and 3) reduced *K* for beetle, but slightly increased *K* for vole and skylark. Randomizing patch arrangement (B to C) decreased *K* for beetle, vole, and skylark. Randomizing patch sizes (C to D) reduced the average size of arable fields, but enlarged field boundaries, hedgerows and roadside verges (Fig. S1). This increased *K* for beetle and vole but decreased *K* for skylark and spider. Randomizing patch sizes changed *K* and *φ* more than the previous landscape modifications for all four species.

**Figure 4:**
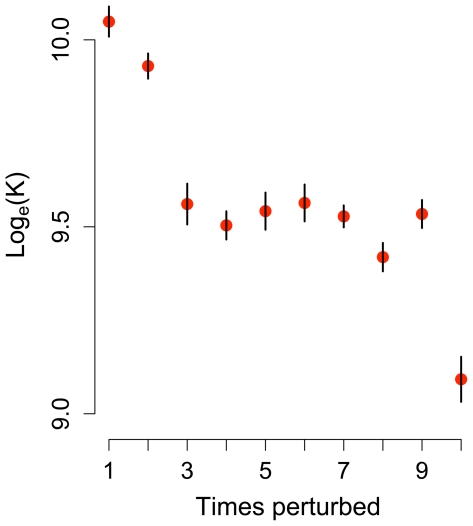
Decrease in equilibrium population size (*K*) for vole with repeated perturbations (mean ±1 SE). Points correspond to asymptotic population sizes for voles in the original landscape, as in Fig. 2, after successive perturbations.

Only the vole population did not recover fully to the original equilibrium between successive strong perturbations (Fig. 4). After the first two strong perturbations *K* decreased abruptly, and then reduced further after the final perturbation.

## Discussion

Landscape modifications that caused reductions in equilibrium population sizes (*K*) resulted in increasing population return times (*φ*) for short-dispersing species as predicted by classical theory. However this was not a simple function of landscape fragmentation. The simulation of beetle populations revealed that *K* decreased when patches became less elongated (A to B) and patch arrangement was randomized (B to C). The most likely reason was that overwintering habitat (e.g. field boundaries [34]) was then displaced from summer habitat (mainly rotational fields). *K* increased when patch sizes were randomized (C to D) because field boundaries were then increased and easier to reach from the fields. Beetle dynamics are thus strongly affected by landscape complementation [8] as both the resources in the fields and in the winter habitat are essential for successful completion of the beetle's life cycle.

Skylark *K* decreased when patch arrangement was randomized because forest patches then became interspersed with agricultural fields, and skylarks avoid nesting close to trees [35]. The far-dispersing opportunistic spider was not affected by patch arrangement or shapes, because neither its ability to disperse nor its local population growth rate depended on the type of the surrounding patches. Both spider and skylark were negatively affected by patch size randomization, which reduced the sizes of the agricultural fields in which these species prosper. Several other studies have found populations to be more strongly affected by a landscape's composition than its configuration (e.g. [2], [5], [6]). Configuration played a minor role in field studies of forest breeding birds across 94 different landscapes [36] and was a relatively poor predictor of occurrence of flying squirrels when compared to landscape composition in a Canadian forest mosaic [37].

It is particularly important to consider population recovery on the landscape scale and to incorporate spatial heterogeneity into population viability analyses (PVAs) if different spatially separated subpopulations respond differently to perturbations. This is the case for the vole, which alone did not recover fully between successive perturbations (Fig. 4). Close examination of the simulation output revealed this was due to local population extinctions in small, isolated grassland patches. Although the interval between successive perturbations was too short to allow isolated patches to be recolonized, it was sufficiently long to allow local populations to recover in larger patches. Interestingly, vole *K* did not always decrease when potential corridors became less elongated (A to B in Fig. 3) as had been predicted on the grounds that voles are short-dispersing species that need corridors to reach good habitat. The simulation output showed that corridors had been transformed to primary habitat (with reduced edge effects [38]) and this affected *K* more strongly than the decreased ability to disperse. Beneficial effects of increased dispersal were also partly counterbalanced by increased dispersal mortality, which may affect population persistence negatively [6]. When patch sizes were randomized (C to D) several of the best vole habitats attained a larger cover (Fig. S1). This had a larger impact on *K* than the other reductions of landscape complexity (A through C).

The landscape we used as a starting point in the simulations (Fig. 1A) was selected because it includes the vast majority of the patch types typical for Danish agricultural landscapes. Our results indicate that landscape composition has a large impact on population dynamics for all four studied species, and it is therefore important to stress that the absolute values of *K* and *φ* would be different in other Danish landscapes. The methods we have applied in this study would, however, also be applicable in other landscapes and for other species.

Several simulation studies have concluded that landscape configuration has little effect on population dynamics (e.g. [2], [5], [6], [39]), or that it is only important for weak dispersers [40]. In contrast our results suggest that configuration is important when the quality of a species' key habitat is affected by the neighboring patches. McIntire et al. [30] found that the persistence of Fender's blue butterflies was promoted by suitably arranging small patches to increase connectivity between populations and in some studies the creation of corridors has resulted in larger population sizes [14]. These studies differ from ours by using simpler representations of the organisms and the landscape. When populations are modeled in an island-like landscape, where animals are unaffected by the surrounding matrix habitat except when dispersing, it will not be possible to detect interactions between specific habitat types as we have done here. Further, models that only include a limited number of patch types are not ideal for comparing effects of landscape modifications across species with different habitat requirements.

The perturbations affected vole and skylark more than spider and beetle. The latter two species have faster life histories (higher *r*_max_) and so recovered faster from low density [41], but at the same time there were large fluctuations in *K* among weather years in these species. This illustrates an important shortcoming of traditional population viability analyses, where the probability of extinction is usually determined from the change in mean population size and its variance [42], [43]. Small, highly variable populations are considered more likely to get below a threshold population size where they go extinct. Our analyses indicate that it may actually be the species with the least variable population sizes (here vole and skylark) that are most at risk. This is most pronounced for the short-dispersing vole that has high *φ* in landscapes where *K* is small. This suggests that currently used PVAs should be supplemented by analyses of the type used here.

Studies of population viability have typically focused on how much habitat is needed to avoid extinction [4] without considering the importance of landscape context. Here we demonstrate that variations in patch shapes, landscape configuration and composition can have pivotal importance for a population's ability to recover after disturbance. Our study is unique in separating the effects of these elements of landscape complexity on population dynamics and in linking them to the ecological mechanisms that control population dynamics [44]. For short dispersing species, such as the vole, whose dynamics are determined by different mechanisms in different parts of the landscape, it will be crucial to discover how spatially separated subpopulations contribute to overall population dynamics.

## Materials and Methods

The simulations were performed in 10×10 km landscapes mapped to a precision of 1 m, containing 18862 patches of 27 different types (Fig. 1). The original landscape is a real agricultural landscape near Bjerringbro in Denmark. We reduced landscape complexity progressively. First we created a landscape with no systematic differences in shapes among the different patch types (‘homogenized shapes’; Fig. 1B). This was done by letting patches grow one m^2^ at a time in random directions, starting at the point where they were centered in the real landscape, and stopping when they reached the size they originally had. The patches' probability of increasing in size were proportional to the fraction they remained to grow; patches that had nearly reached the size they had in the original landscape therefore grew slowly. Secondly, we randomized the patch arrangement by interchanging patches of the same size at random (Fig. 1C). Only patch types were interchanged; outlines were retained as in Fig. 1B. When several fields are located next to each other they cannot be distinguished on Fig. 1B, even though their suitability for the modeled species depended on the crops grown on them. Finally, starting with the landscape in Fig. 1C, we randomly interchanged patches irrespective of their size class, thereby creating a landscape where the total cover of different patch types was proportional to their frequency (Figs. 1D; S2). Roads, rivers and houses that acted as dispersal barriers to some species were left untouched in all landscapes. This constrained the growth of individual patches.

Four species with complementary dispersal and reproductive rates were selected for study: a ground beetle (*Bembidion lampros*), field vole (*Microtus agrestis*), skylark (*Alauda arvensis*), and a linyphiid spider (*Erigone atra*). The first two species are short dispersing; beetle and spider are short-lived and have high reproductive rates. The beetle is a flightless species associated with agricultural fields. It depends on vegetated field boundaries for winter hibernation. Field voles are predominately associated with unmanaged grasslands, and when animals move to other habitat types this affects their behavior, mortality and reproduction. Skylarks nest and feed in open fields and field margins. Their reproductive success depends on the food acquisition rate of the adults, which in turn depends on patch type, weather etc. The spider is associated with agricultural fields. It is able to disperse far by ballooning, but this results in high mortality.

The study species were modeled using four realistic IBMs [32] in which each individual's movement, growth, fecundity, dispersal and the risk of dying depended on which patch type it was located in, daily weather, farming practices, interactions with other individuals, its experience and physiological state (c.f. [33], [45], [46]). Details of the models are provided elsewhere for beetle [47], [48], field vole [32], [48], [49], skylark [50], [51] and spider [34], [52]. The development and parameterization of our IBMs followed the ideas formulated in the Pattern Oriented Modeling strategy [46], [53], and models were successively improved and reparameterized until good fits between emergent patterns and independent field data were obtained. All models were based on the same underlying dynamic landscape model where growth of different crop types etc. reflects daily changes in farming activities and weather [32]. Model documentation following a modified version of the ODD protocol [54] is available in [55]. The same models and species were used by Sibly et al. [56], who focused on spatial variations in unperturbed populations. The population dynamics in the four models (here quantified by *K* and *φ*) were emergent properties, i.e. they were determined indirectly through the effect that local environmental conditions had on the behavior of each individual. The models' ability to generate several close-to-natural emergent population patterns makes them substantially different from other models that have been used for investigating effects of landscape structure [14], [15], [30], [31].

The overall dynamics of IBMs are most strongly influenced by variables that have a strong effect on fitness [33]. Inclusion of additional variables in a model can make it more mechanistically realistic and improve the match between model predictions and population patterns observed in nature (e.g. variations in population size in space and time). The aim in the models we used was to obtain as close a fit as possible between emergent patterns and real-world data by including all available information about variables that were known to influence individual behavior. The mechanisms that controlled population behavior in our model species are representative of a wide range of species, which suggests that the conclusions we reached should generalize to other species and landscapes.

Population size (*N*_t_) was recorded yearly for 170 simulation years (Fig. S2), allowing populations to recover 10 times from disturbances. The effect of running the model repeatedly on replicate landscapes was explored in Fig. S3. The simulations indicated that our results are robust when landscapes are repeatedly simplified using the methods presented here. 1990s weather data were used sequentially to calculate daily vegetation growth etc. Populations were disturbed by removing 80% or 95% of all individuals at random every 17th year (Fig. S2). Increases in *N*_t_ with time after disturbance were modeled as logistic, following e.g. Sæther *et al.* [23]. For beetle, vole and spider we used three-parameter logistic models to describe return to equilibrium:
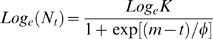
(1)


Here *K* is the equilibrium population size, *m* is the inflection point (the value of *t* corresponding to *Log_e_*(*K*)/2), and *φ* is the ‘shape parameter’. Small values of *φ* indicate that the population returns swiftly to equilibrium.

In our simulations crop growth, farming practices and behavior of individual animals were affected by the weather. This produced variation in *K* and *φ* among weather years *y*. The logistic models were therefore fitted using non-linear mixed models in R 2.6.2 [57] using the discrete variable *y* as a random grouping variable indicating weather year (see [58] for details). Equilibrium population sizes for unperturbed populations were modeled using the linear model 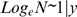 where the intercept varied among weather years, but no slope parameter was included. Within-group errors were uncorrelated, homogeneous and normally distributed. For the skylark, population growth rate initially increased with time after disturbance, and a four-parameter logistic equation was used to obtain a better fit. *K* and *φ* were estimated from asymptote and shape parameters for different weather years using nonlinear mixed models. In total 48 models were analyzed ( = 4 spp ×3 perturbation intensities ×4 landscapes).

## Supporting Information

Appendix S1 Relationship between return time and return rate.
(0.15 MB PDF)

Figure S1 Land cover in the 10×10-km Bjerringbro landscape. Size class distribution for selected patch types for (A) landscapes A–C and (B) landscape D in Figs. 1 and 3. Patches were divided in classes of size Log_10_(*x*)/4 where *x* is patch size in m^2^. Areas of circles are proportional to the number of patches in a size class. Buildings, lakes, streams, roads and railways (red circles) were left untouched by all patch randomizations. Numbers in right hand side of the figure give mean patch size in hectares (ha).
(1.46 MB TIF)

Figure S2 Monthly population sizes for vole. Population sizes during the first 44 years of a 181-year simulation (example). The first 11 y were used as a burn-in period and only data from the last 170 years were analyzed. Only population sizes from 1 January were used for fitting logistic growth curves. The illustrated populations were perturbed by 95% every 17 y (dashed vertical lines). Different colors indicate landscapes of different complexities.
(0.81 MB TIF)

Figure S3 Variations in *K* and *ϕ* among replicate landscapes. For each of the landscape types B–D we generated 10 landscapes; each of these were used in a single 181-year simulation for the studied species. The grey circles show *K* and *ϕ* for each landscape (calculated as in Figs 2–3), and error bars show the 95% confidence intervals corresponding to these. Variations in *K* and *ϕ* result from differences among landscapes and stochastic variations among simulations. Results are only shown for vole and skylark, which were relatively strongly influenced by differences among landscapes.
(2.58 MB TIF)
