# Supplementary material for: The Effects of Landscape Modifications on the Long-Term Persistence of Animal Populations
Source: PLoS One. 2010 Jan 28;5(1):e8932. doi: 10.1371/journal.pone.0008932 (PMC2812489; doi:10.1371/journal.pone.0008932)
Supplement: Appendix S1 — Relationship between return time and return rate. (0.15 MB PDF) [file pone.0008932.s001.pdf]

The variable ϕ that we use in this study is equivalent to the reciprocal of return rate as defined by May [1] and is thus a measure of return time (proof follows). Return rate is sometimes referred to as the strength of density dependence [2,3]. May [1] defines return rate as

$$\textit{return rate} \equiv - [\mathrm{d}pgr/\mathrm{d}Log_e N]_K \equiv - [N\ \mathrm{d}pgr/\mathrm{d}N]_K \tag{2}$$

where *pgr* is the population growth rate. Note that

$$pgr = \frac{1}{N}\frac{dn}{dt} = \frac{d(Log_e N)}{dt} = \frac{Log_e K \exp[(m-t)/\phi]}{\phi(1+\exp[(m-t)/\phi]^2} \tag{3}$$

Now use (1) to substitute into (3) to get

$$= \frac{(Log_e N)^2 (Log_e K / Log_e N - 1)}{\phi Log_e K} \tag{4}$$

Writing $x = Log_e N$ gives

$$pgr = \frac{x^2 (Log_e K / x - 1)}{\phi Log_e K} = \frac{x}{\phi} - \frac{x^2}{\phi Log_e K} \tag{5}$$

By definition *return rate* = - $[\mathrm{d}pgr/\mathrm{d}x]_K = -\frac{1}{\phi} + \left[\frac{2x}{\phi Log_e K}\right]_K = \frac{1}{\phi}$ (6)

Note that the May definition was designed for discrete generation models in which *pgr* is related to *N* in the previous time unit. Applying this formulation to the fitted logistic equation is assuming that *pgr* is related to current *N*, cf. Sibly *et al.* [4].

SI References

1. May RM (1975) Biological populations obeying difference equations: stable points, stable cycles, and chaos. J Theor Biol 51: 511-524.
2. Lande R, Engen S, Sæther BE, Coulson T (2006) Estimating density dependence from time series of population age structure. Am Nat 168: 76-87.
3. Sæther B-E, Engen S (2002) Pattern of variation in avian population growth rates. Philos Trans R Soc Lond B Biol Sci 357: 1185-1195.
4. Sibly RM, Barker D, Hone J, Pagel M (2007) On the stability of populations of mammals, birds, fish and insects. Ecol Lett 10: 970-976.
