# Supplementary figures and images for: The Effects of Landscape Modifications on the Long-Term Persistence of Animal Populations
Source: PLoS One. 2010 Jan 28;5(1):e8932. doi: 10.1371/journal.pone.0008932 (PMC2812489; doi:10.1371/journal.pone.0008932)

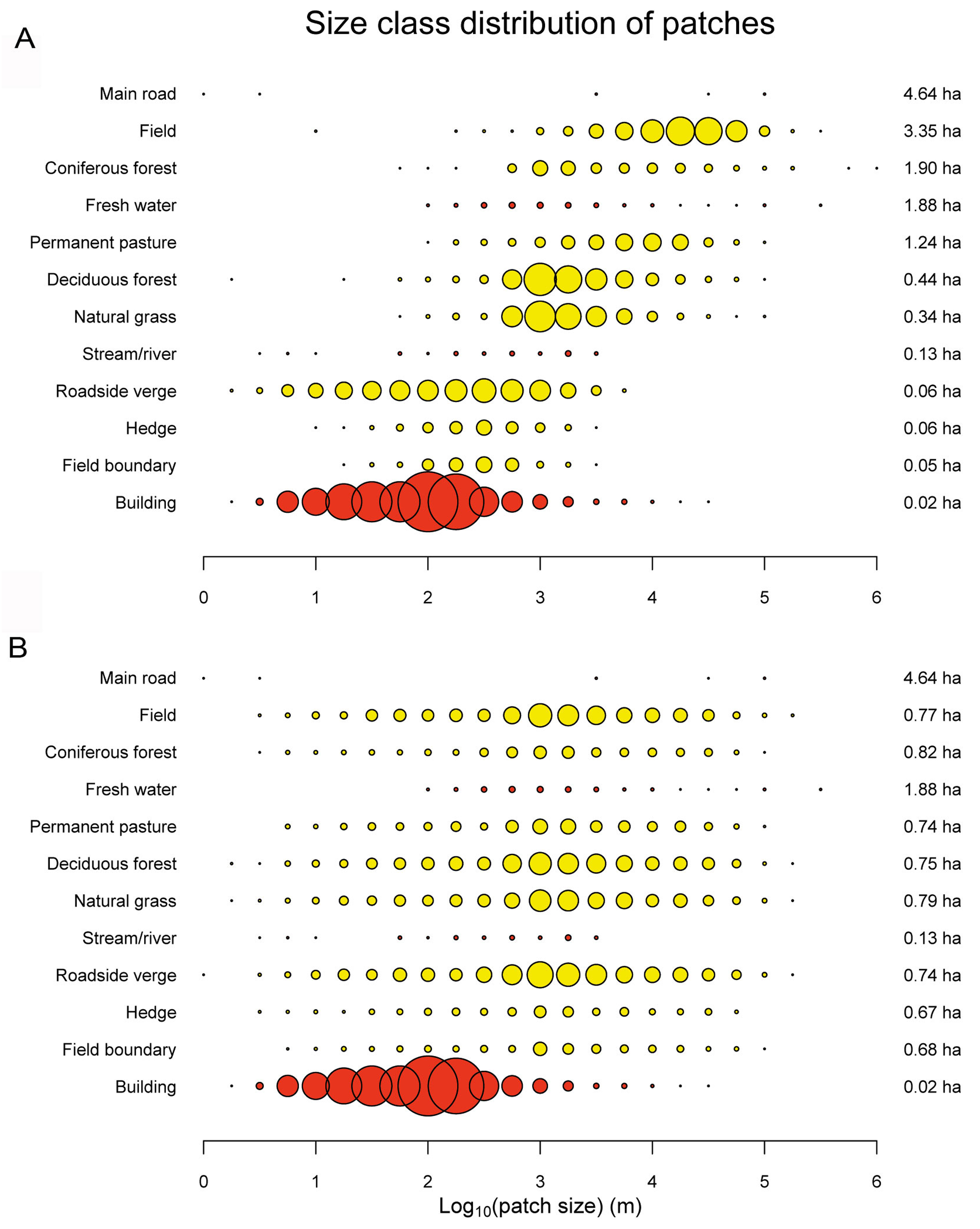

Supplement: Figure S1 — Land cover in the 10×10-km Bjerringbro landscape. Size class distribution for selected patch types for (A) landscapes A–C and (B) landscape D in Figs. 1 and 3. Patches were divided in classes of size Log10(x)/4 where x is patch size in m2. Areas of circles are proportional to the number of patches in a size class. Buildings, lakes, streams, roads and railways (red circles) were left untouched by all patch randomizations. Numbers in right hand side of the figure give mean patch size in hectares (ha). (1.46 MB TIF) [file pone.0008932.s002.tif]

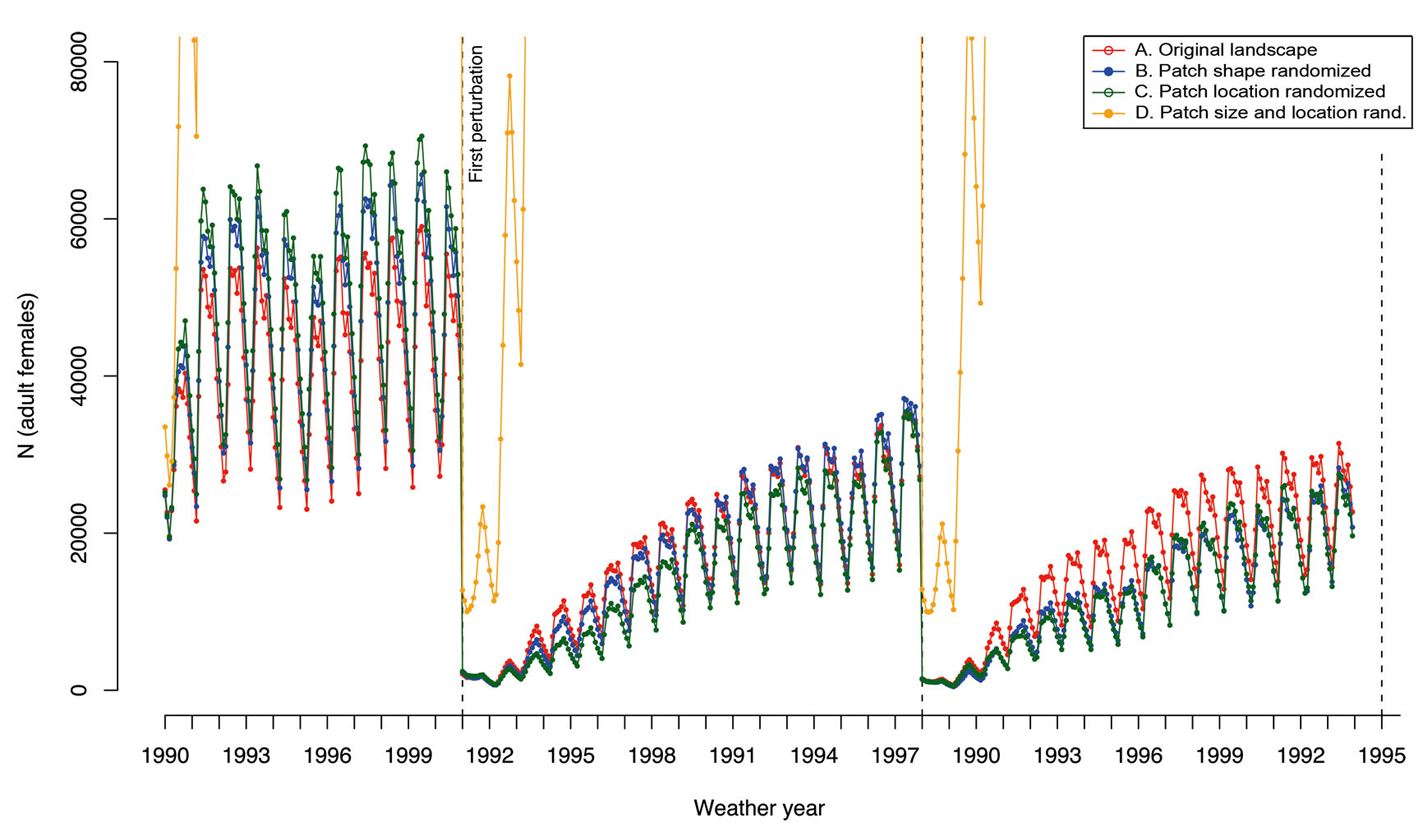

Supplement: Figure S2 — Monthly population sizes for vole. Population sizes during the first 44 years of a 181-year simulation (example). The first 11 y were used as a burn-in period and only data from the last 170 years were analyzed. Only population sizes from 1 January were used for fitting logistic growth curves. The illustrated populations were perturbed by 95% every 17 y (dashed vertical lines). Different colors indicate landscapes of different complexities. (0.81 MB TIF) [file pone.0008932.s003.tif]
